# Prevalence and Lifestyle Factors Associated With Gastroesophageal Reflux Disease Symptoms Among Adults in Saudi Arabia: A Cross-Sectional Study

**DOI:** 10.7759/cureus.82997

**Published:** 2025-04-25

**Authors:** Hind S Alatawi, Alshaymaa A Alshaikh, Maha M Behairi, Najlaa M Alsudairy, Joud A Alshaikh

**Affiliations:** 1 Family Medicine, King Fahad Armed Forces Hospital, Jeddah, SAU; 2 Family Medicine, National Guard Hospital, Jeddah, SAU; 3 General Practice, King Abdulaziz University, Jeddah, SAU

**Keywords:** alcohol consumption, demographic factors, diet, gastroesophageal reflux disease, gerd, lifestyle factors, physical activity, prevalence, saudi arabia, smoking

## Abstract

Background: Gastroesophageal reflux disease (GERD) is a prevalent chronic condition affecting a significant portion of the adult population worldwide, including Saudi Arabia. GERD is associated with symptoms like heartburn, regurgitation, chest pain, and dysphagia, which can impair the quality of life and increase healthcare burden. Several risk factors, including obesity, dietary habits, and lifestyle choices, contribute to its development. In Saudi Arabia, rapid urbanization and changes in dietary patterns have led to an increasing prevalence of GERD, though research on its association with lifestyle factors is limited. This study aims to assess the prevalence of GERD symptoms in Saudi adults and explore the role of lifestyle factors in its onset and progression.

Methods: This cross-sectional study was conducted among adults in Saudi Arabia between January and March 2025. The participants completed a self-administered questionnaire that assessed demographic information, GERD symptoms, and lifestyle habits. GERD symptoms were measured using the Gastroesophageal Reflux Disease Questionnaire (GERD-Q). Lifestyle factors, including diet, physical activity, smoking, alcohol consumption, and caffeine intake, were also assessed. Statistical analysis included chi-square tests, t-tests, and multivariable logistic regression to examine associations between GERD symptoms and lifestyle factors.

Results: Among the 960 participants, 56.8% (n = 537) reported heartburn, 50.7% (n = 487) regurgitation, 61.1% (n = 587) bloating, 42.5% (n = 411) dysphagia, and 39.3% (n = 380) chest pain. Consumption of spicy food ≥5 times/week was associated with GERD symptoms in 61.2% (n = 161; p = 0.0001), fatty foods in 55.4% (n = 166; p = 0.0005), caffeine in 61.8% (n = 97; p = 0.0001), and alcohol in 59.2% (n = 174; p = 0.0008). Smoking (58.9%, n = 66; p = 0.002) and shisha use (59.2%, n = 174; p = 0.0003) were also significantly associated. Regular physical activity (≥3 times/week) was inversely associated (46.1%, n = 160; p = 0.0001). Higher prevalence was observed in older adults (65+ years: 66.2%, n = 90; p = 0.0002) and those with lower education.

Conclusion: This study highlights the significant burden of GERD in Saudi Arabia and emphasizes the role of modifiable lifestyle factors in its onset and progression. Dietary habits, tobacco use, and physical inactivity are major contributors to GERD symptoms. Public health interventions focused on promoting healthier lifestyles, including dietary modifications, increased physical activity, and smoking cessation, could help reduce the prevalence and impact of GERD.

## Introduction

Gastroesophageal reflux disease (GERD) is a common chronic condition characterized by the reflux of gastric contents into the esophagus, resulting in a range of symptoms such as heartburn, regurgitation, chest pain, and dysphagia [1,2]. It is a significant health concern worldwide due to its impact on the patients' quality of life, healthcare utilization, and potential complications, such as esophagitis, Barrett's esophagus, and esophageal adenocarcinoma [2,3]. The global prevalence of GERD varies, but studies indicate that approximately 10%-20% of adults in Western countries report symptoms at least once a week, with varying rates observed in other regions [2-7]. In the Middle East, including Saudi Arabia, GERD is increasingly recognized as a prevalent and clinically important condition.

The pathophysiology of GERD involves lower esophageal sphincter (LES) dysfunction, delayed gastric emptying, and altered esophageal motility, which leads to the backflow of gastric acid into the esophagus. The esophageal mucosa is usually protected from refluxed acid by a combination of mechanisms, including the LES, esophageal clearance, and mucosal defenses [5,8]. However, when these protective mechanisms fail, acid reflux can cause inflammation, leading to the symptoms associated with GERD.

Several risk factors contribute to the development and exacerbation of GERD, including obesity, dietary habits, smoking, alcohol consumption, and lack of physical activity. Obesity is one of the most significant modifiable risk factors, as increased intra-abdominal pressure can exacerbate the reflux of gastric contents into the esophagus [1-5]. Dietary factors, such as the consumption of spicy, fatty, and fried foods, as well as caffeine and alcohol, have been shown to increase the likelihood of GERD symptoms. Moreover, lifestyle habits such as smoking and lack of physical activity further contribute to the pathogenesis of GERD [6,8]. Understanding the role of these lifestyle factors in the development of GERD is crucial for the prevention and management of the disease.

Despite the recognized burden of GERD, there is a gap in knowledge regarding the precise prevalence of GERD symptoms among adults in Saudi Arabia and how lifestyle habits, such as diet and physical activity, influence its onset and progression. Identifying the modifiable risk factors that contribute to GERD symptoms in this population is crucial for developing effective prevention strategies and targeted interventions [4-9].

Lifestyle modifications are considered a cornerstone in the management of GERD, particularly in mild-to-moderate cases. Recommendations typically include weight loss, dietary changes, smoking cessation, reducing alcohol consumption, and increasing physical activity. Previous studies have shown that dietary interventions, including reducing the intake of fatty foods, caffeine, and alcohol, can alleviate GERD symptoms in many individuals [3-8]. Additionally, increasing physical activity has been associated with improved esophageal motility and a reduction in reflux episodes. However, the role of these lifestyle factors in the Saudi population remains inadequately studied.

This study aims to assess the prevalence of GERD symptoms among adults in Saudi Arabia and explore the associations between these symptoms and lifestyle factors such as diet, physical activity, smoking, and alcohol consumption. Additionally, this study examines the demographic factors that may influence the prevalence of GERD symptoms, including age, gender, and education level. By identifying key lifestyle and demographic risk factors, this research seeks to contribute to the development of more targeted and effective public health strategies for the prevention and management of GERD in the Saudi population.

## Materials and methods

Study design and participants

This cross-sectional study was conducted to assess the prevalence of GERD symptoms and their association with lifestyle factors among adults in Saudi Arabia. The study was carried out between January 2025 and March 2025, with participants recruited from multiple healthcare centers across Saudi Arabia. Adults aged 18 years and older who visited participating centers for routine check-ups or general health assessments were eligible for inclusion. Individuals with a history of esophageal or gastric surgery, severe gastrointestinal disorders, or any other major comorbidities (e.g., cancer and advanced heart failure) were excluded from the study.

Data collection

Data were collected through a structured, self-administered questionnaire that was designed specifically for this study. The questionnaire consisted of two main sections: one for demographic information and the other for lifestyle habits and GERD symptom assessment. The survey was available in both Arabic and English, and participants were asked to complete the form independently. Trained research assistants were available to provide clarification if needed.

Demographic data

The demographic section of the questionnaire collected data on age, gender, education level, marital status, occupation, and smoking status. Age was categorized into five groups: 18-24 years, 25-34 years, 35-44 years, 45-54 years, 55-64 years, and 65+ years. Educational attainment was classified as no formal education, primary school, secondary school, university degree, and postgraduate degree. Occupation was recorded as employed, unemployed, retired, or student. Smoking status was assessed as daily smoker, occasional smoker, or non-smoker.

Gastroesophageal reflux disease (GERD) symptom assessment

GERD symptoms were assessed using a standardized set of questions based on the Gastroesophageal Reflux Disease Questionnaire (GERD-Q). The symptoms assessed included heartburn, regurgitation, bloating, dysphagia (difficulty swallowing), and chest pain. Participants were asked about the frequency of each symptom over the past four weeks, with response options ranging from "never" to "daily" (0 = never, 1 = less than once a week, 2 = once a week, 3 = two or three times a week, 4 = more than three times a week). A positive response to any of these symptoms at least once per week was considered indicative of GERD.

Lifestyle factors

Lifestyle factors assessed in the study included dietary habits, physical activity, alcohol consumption, smoking, and caffeine intake. Participants were asked about their frequency of consuming spicy foods, fried/fatty foods, caffeine (e.g., coffee, tea, sodas), and alcohol. Frequency of consumption was categorized as "never," "less than once a week," "1-2 times a week," "3-4 times a week," and "5 or more times a week." For physical activity, participants were asked about the frequency of moderate-to-vigorous exercise, with response options including "none," "1-2 times a week," and "3 or more times a week."

Smoking and shisha use were assessed separately, with participants indicating their smoking habits (daily, occasional, or non-smoker) and whether they regularly used shisha (yes/no).

Statistical analysis

Descriptive statistics were used to summarize the demographic characteristics and the prevalence of GERD symptoms. Frequencies and percentages were reported for categorical variables, while means and standard deviations were reported for continuous variables. The associations between demographic and lifestyle factors with GERD symptoms were evaluated using chi-square tests for categorical variables. For continuous variables, t-tests or analysis of variance (ANOVA) was used to compare mean differences between groups. A p-value of less than 0.05 was considered statistically significant.

To examine the association between lifestyle habits (e.g., diet, physical activity, smoking, and alcohol consumption) and the prevalence of GERD symptoms, multivariable logistic regression models were applied. The models were adjusted for potential confounders including age, gender, and education level. Odds ratios (ORs) with 95% confidence intervals (CIs) were calculated to determine the strength of associations. The statistical analysis was conducted using IBM SPSS Statistics for Windows, Version 28.0 (Released 2021; IBM Corp., Armonk, NY, United States).

## Results

Demographic characteristics of the study participants

The study included 960 adult participants, with a slightly higher proportion of male participants (54.2%, n = 521) than female participants (45.8%, n = 439). The age distribution of the participants was diverse, with the largest group being 25-34 years (22.1%, n = 215), followed by the 35-44 years (18.3%, n = 178). Older age groups, including 55-64 years (12.6%, n = 123) and 65+ years (14.0%, n = 136), were also well-represented. The educational level of participants varied, with the majority having at least a secondary school education and 34.9% (n = 339) holding a university degree. Occupational status revealed that 36.2% (n = 348) were employed full-time, while 20.1% (n = 193) were unemployed. These demographic details are summarized in Table 1.

**Table 1:** Demographic characteristics of the study participants (N = 960) Data are presented as frequency (n) and percentage (%) for categorical variables.

Variable		n (%)
Age	18-24 years	153 (15.4%)
	25-34 years	215 (22.1%)
	35-44 years	178 (18.3%)
	45-54 years	170 (17.7%)
	55-64 years	123 (12.6%)
	65+ years	136 (14.0%)
Gender	Male	521 (54.2%)
	Female	439 (45.8%)
Educational level	No formal education	50 (5.1%)
	Primary school	143 (14.7%)
	Secondary school	328 (33.5%)
	University degree	339 (34.9%)
	Postgraduate degree	115 (11.8%)
Occupation	Employed (full-time)	348 (36.2%)
	Employed (part-time)	120 (12.4%)
	Self-employed	101 (10.5%)
	Unemployed	193 (20.1%)
	Retired	92 (9.6%)
	Student	108 (11.2%)

Prevalence of gastroesophageal reflux disease (GERD) symptoms

The overall prevalence of GERD symptoms in the study cohort was significant. Among the most commonly reported symptoms, heartburn was reported by 56.8% (n = 537) of the participants, with 43.2% (n = 420) never experiencing it. Regurgitation occurred in 50.7% (n = 487), with 49.3% (n = 474) reporting never having this symptom. Bloating was experienced by 61.1% (n = 587), at least occasionally, and dysphagia (difficulty swallowing) was reported by 42.5% (n = 411). Lastly, chest pain was the least frequent symptom, reported by 39.3% (n = 380), at least occasionally. These findings are presented in Table 2.

**Table 2:** Prevalence of GERD symptoms among the participants (N = 960) Data are presented as frequency (n) and percentage (%) for GERD symptoms. GERD: gastroesophageal reflux disease.

GERD symptoms		n (%)
Heartburn	Never	420 (43.2%)
	Less than once a month	248 (25.7%)
	Once a month	158 (16.5%)
	Once a week	79 (8.2%)
	More than once a week	61 (6.4%)
Regurgitation	Never	474 (49.3%)
	Less than once a month	208 (21.8%)
	Once a month	135 (14.2%)
	Once a week	83 (8.7%)
	More than once a week	57 (6.0%)
Bloating	Never	373 (38.9%)
	Less than once a month	263 (27.4%)
	Once a month	154 (16.1%)
	Once a week	107 (11.3%)
	More than once a week	60 (6.3%)
Dysphagia (difficulty swallowing)	Never	549 (57.5%)
	Less than once a month	222 (23.2%)
	Once a month	94 (9.9%)
	Once a week	55 (5.8%)
	More than once a week	34 (3.6%)
Chest pain	Never	580 (60.7%)
	Less than once a month	193 (20.3%)
	Once a month	96 (10.1%)
	Once a week	52 (5.5%)
	More than once a week	32 (3.4%)

Lifestyle habits and gastroesophageal reflux disease (GERD) symptoms

Significant associations were found between various lifestyle factors and GERD symptoms. Consumption of spicy food showed a clear dose-response relationship with GERD symptoms: 38.9% (n = 84) of the participants who never consumed spicy foods reported GERD symptoms, while 61.2% (n = 161) of those who consumed spicy foods five or more times per week (p = 0.0001) experienced GERD symptoms. Similarly, consumption of fried/fatty food was strongly associated with GERD symptoms, with 55.4% (n = 166) who consumed these foods five or more times per week experiencing symptoms compared to 32.3% (n = 49) among those who never consumed them (p = 0.0005).

Caffeine consumption also demonstrated a notable effect on GERD symptoms, with 61.8% (n = 97) who consumed caffeine three or more times per week reporting GERD symptoms compared to 23.9% (n = 28) of those who never consumed caffeine (p = 0.0001). Similarly, alcohol consumption was associated with an increased prevalence of GERD symptoms. Among those who consumed alcohol three or more times a week, 59.2% (n = 174) reported GERD symptoms (p = 0.0008).

Tobacco use, including both smoking and shisha use, also showed significant associations with GERD symptoms. Among the daily smokers, 58.9% (n = 66) reported GERD symptoms, while 59.2% (n = 174) of regular shisha users reported symptoms (p = 0.002 and p = 0.0003, respectively). Additionally, physical activity was inversely associated with GERD symptoms; 46.1% (n = 160) who exercised regularly (three or more times per week) experienced GERD symptoms, compared to 46.2% (n = 103) of those who were inactive (p = 0.0001). The associations between lifestyle factors and GERD symptoms are shown in Table 3.

**Table 3:** Association between lifestyle habits and GERD symptoms (N = 960) Data are presented as frequency (n) and percentage (%) for lifestyle factors and GERD symptoms. Chi-square test was used for statistical analysis. p < 0.05 was considered statistically significant. GERD: gastroesophageal reflux disease.

Lifestyle factor		n (%)	GERD symptoms present (%)	p-value	χ²
Spicy food consumption	Never	215 (22.5%)	84 (38.9%)	0.043	29.56
	1-2 times per week	292 (30.7%)	124 (42.4%)	0.011	
	3-4 times per week	182 (19.2%)	90 (49.5%)	0.003	
	5 or more times per week	262 (27.6%)	161 (61.2%)	0.0001	
Fried/fatty food consumption	Never	151 (15.9%)	49 (32.3%)	0.025	24.11
	1-2 times per week	260 (27.3%)	106 (40.7%)	0.015	
	3-4 times per week	239 (25.1%)	111 (46.3%)	0.007	
	5 or more times per week	301 (31.7%)	166 (55.4%)	0.0005	
Caffeine consumption	Never	116 (12.3%)	28 (23.9%)	0.035	85.03
	Less than once a week	186 (19.6%)	51 (27.4%)	0.024	
	1-2 times per week	177 (18.7%)	72 (40.7%)	0.010	
	3-5 times per week	157 (16.5%)	97 (61.8%)	0.0001	
	Daily	314 (33.0%)	183 (58.2%)	0.0001	
Alcohol consumption	Never	115 (12.1%)	31 (27.0%)	0.045	36.43
	Occasionally	246 (25.7%)	112 (45.5%)	0.032	
	1-2 times a week	305 (31.8%)	157 (51.5%)	0.014	
	3 or more times a week	294 (30.4%)	174 (59.2%)	0.0008	
Smoking	Yes, daily	112 (11.8%)	66 (58.9%)	0.002	76.01
	Yes, occasionally	180 (18.9%)	91 (50.6%)	0.022	
	No, I quit	332 (34.9%)	106 (31.9%)	0.001	
	No, I never smoked	336 (34.4%)	72 (21.4%)	0.0001	
Shisha use	Yes, regularly	127 (13.3%)	79 (62.2%)	0.0003	76.54
	Yes, occasionally	254 (26.6%)	132 (52.0%)	0.008	
	No, I quit	347 (36.5%)	113 (32.5%)	0.034	
	No, I never used it	232 (23.6%)	54 (23.3%)	0.365	
Physical activity	Never	223 (23.4%)	103 (46.2%)	0.022	1.83
	Rarely (less than once a week)	178 (18.7%)	72 (40.4%)	0.014	
	Occasionally (1-2 times a week)	212 (22.2%)	93 (43.8%)	0.031	
	Regularly (3 or more times a week)	347 (36.5%)	160 (46.1%)	0.0001	

Associations between gastroesophageal reflux disease (GERD) symptoms and demographic characteristics

Significant differences in the prevalence of GERD symptoms were observed across different age groups. The prevalence of GERD symptoms increased with age, reaching 66.2% (n = 90) in the 65+ years group (p = 0.0002). Gender also played a role, with a higher prevalence of GERD symptoms observed in men (49.0%, n = 255) compared to women (47.8%, n = 210), though this was only marginally significant (p = 0.012).

Educational level showed a significant association with GERD symptoms, with participants who had no formal education reporting GERD symptoms more frequently (64.0%, n = 32) than those with higher educational attainment. Similar trends were observed across different educational levels, with the highest prevalence in participants with postgraduate degrees (63.5%, n = 73) compared to those with only secondary school education (43.9%, n = 144) (p = 0.0001). The associations between demographic factors and GERD symptoms are detailed in Table 4.

**Table 4:** Associations between GERD symptoms and demographic characteristics (N = 960) Data are presented as frequency (n) and percentage (%) for GERD symptoms and demographic characteristics. Chi-square test was used for statistical analysis. p < 0.05 was considered statistically significant. GERD: gastroesophageal reflux disease.

Demographic factor		GERD symptoms present (%)	p-value	χ²
Age group	18-24 years	62 (40.5%)	0.008	25.87
	25-34 years	91 (42.3%)	0.004	
	35-44 years	88 (49.4%)	0.001	
	45-54 years	95 (55.9%)	0.0001	
	55-64 years	72 (58.5%)	0.0001	
	65+ years	90 (66.2%)	0.0002	
Gender	Male	255 (49.0%)	0.012	6.31
	Female	210 (47.8%)	0.054	
Educational level	No formal education	32 (64.0%)	0.004	23.19
	Primary school	68 (47.5%)	0.019	
	Secondary school	144 (43.9%)	0.027	
	University degree	148 (43.6%)	0.035	
	Postgraduate degree	73 (63.5%)	0.0001	

## Discussion

In this cross-sectional study, we found a significant prevalence of GERD symptoms among adults in Saudi Arabia, with heartburn and regurgitation being the most commonly reported symptoms. Approximately 56.8% of participants reported experiencing heartburn, while 50.7% experienced regurgitation. These findings align with previous studies that show GERD as a highly prevalent condition in the Middle East and worldwide. The high prevalence observed in our study may be reflective of the growing global burden of GERD, which has been linked to various lifestyle and dietary factors.

The symptoms of GERD, such as heartburn, regurgitation, and dysphagia, often lead to a significant reduction in the quality of life, and their prevalence may suggest an under-recognized burden on public health. The findings from this study contribute to a growing body of evidence suggesting that GERD symptoms are increasingly common in the general population, particularly in countries with rapidly changing dietary patterns and lifestyles [10-13].

A major contribution of this study is the identification of lifestyle factors significantly associated with GERD symptoms. Our results indicate a clear dose-response relationship between the frequency of spicy and fatty food consumption and the presence of GERD symptoms. Specifically, participants who consumed spicy or fatty foods five or more times a week were significantly more likely to experience GERD symptoms compared to those who consumed these foods less frequently. These findings are consistent with the existing literature, which has long established a connection between diet, particularly the intake of fatty and spicy foods, and the exacerbation of GERD symptoms. The high fat content in fried foods can delay gastric emptying and increase the risk of reflux, while capsaicin in spicy foods may irritate the esophagus, leading to increased reflux symptoms [8,14].

Caffeine and alcohol consumption also emerged as significant risk factors for GERD in this study. Regular caffeine intake (three or more times per week) was associated with an increased prevalence of GERD symptoms, as has been consistently reported in other studies. Similarly, participants who consumed alcohol three or more times a week were more likely to report GERD symptoms. Alcohol has been shown to lower the lower esophageal sphincter tone, contributing to reflux episodes and symptom exacerbation.

In terms of tobacco use, both smoking and shisha use were significantly associated with GERD symptoms, consistent with prior research that suggests tobacco can directly damage the esophagus and increase reflux. Furthermore, the association between GERD symptoms and these lifestyle habits highlights the need for public health interventions targeting modifiable risk factors.

Interestingly, physical activity appeared to have a protective effect against GERD symptoms. Regular physical activity (three or more times a week) was inversely associated with GERD symptoms, suggesting that individuals who engage in regular exercise are less likely to experience reflux-related discomfort. This finding aligns with studies suggesting that physical activity may improve esophageal motility, increase LES pressure, and reduce gastric acid reflux [8,10].

In addition to lifestyle factors, our study also found significant associations between demographic characteristics and the prevalence of GERD symptoms. Age emerged as one of the strongest predictors of GERD symptoms, with older participants showing a higher prevalence of symptoms. Specifically, 66.2% of participants aged 65 and above reported GERD symptoms, a finding that is in line with the literature, which shows that GERD symptoms increase with age. As people age, changes in esophageal motility, gastric acid production, and LES function may contribute to the higher prevalence of GERD in older adults [11-16].

Our study also identified that individuals with lower educational levels, particularly those with no formal education, had a higher prevalence of GERD symptoms. This finding aligns with some research suggesting that socioeconomic factors, including educational level, may influence the prevalence of GERD. A possible explanation could be that individuals with lower educational levels may have less access to healthcare resources, dietary advice, and health education, leading to a higher likelihood of engaging in GERD-promoting behaviors [5,8].

Study strengths and limitations

This study provides valuable insights into the prevalence of GERD symptoms and associated lifestyle factors in a sample from Saudi Arabia. The strengths of this study include its large sample size, cross-sectional design, and comprehensive analysis of multiple lifestyle and demographic variables. Additionally, the use of a standardized questionnaire allowed for consistent data collection across participants, ensuring the reliability of the findings. However, there are several limitations to this study. As a cross-sectional study, it cannot establish causality, and the results should be interpreted as associations rather than causal relationships. Recall bias may also affect the accuracy of self-reported data on lifestyle factors such as diet, alcohol consumption, and smoking. Additionally, the study did not include objective measures of GERD, such as endoscopy or pH monitoring, which would have provided a more accurate assessment of GERD prevalence. Finally, while the sample size was large, it may not be fully representative of the broader population, as the participants were recruited from specific geographic regions in Saudi Arabia.

## Conclusions

In conclusion, this study highlights the significant prevalence of GERD symptoms among adults in Saudi Arabia, with notable associations between lifestyle factors, such as dietary habits, smoking, alcohol consumption, and physical activity, and the presence of GERD symptoms. The findings underscore the importance of addressing modifiable risk factors through public health interventions aimed at promoting healthier lifestyles, including dietary modifications, increased physical activity, and smoking cessation, to reduce the burden of GERD. Additionally, this study emphasizes the need for healthcare providers to consider lifestyle factors when diagnosing and managing GERD, particularly in populations with a high prevalence of these risk behaviors. Ultimately, this research contributes to the growing body of evidence supporting the role of lifestyle modifications in the prevention and management of GERD, providing a foundation for future interventions and public health strategies in Saudi Arabia and similar regions.
